# DMM Outstanding Paper Prize 2023 winners: Lídia Faria, Ffion R. Hammond and Amy Lewis

**DOI:** 10.1242/dmm.050893

**Published:** 2024-06-05

**Authors:** Rachel Hackett

**Affiliations:** The Company of Biologists, Bidder Building, Station Road, Cambridge CB24 9LF, UK

## Abstract

Disease Models & Mechanisms (DMM) is delighted to announce that the winners of the DMM Outstanding Paper Prize 2023 are Lídia Faria for their Research Article (titled ‘Activation of an actin signaling pathway in pre-malignant mammary epithelial cells by P-cadherin is essential for transformation’), and Ffion R. Hammond and Amy Lewis for their Resource Article (titled ‘An *arginase 2* promoter transgenic line illuminates immune cell polarisation in zebrafish’). The two prizes of £1000 are awarded to the first author(s) of the papers that are judged by the journal's Editors to be the most outstanding contribution to the journal that year.

## Teaser

**Summary:** DMM announces the winners of the DMM Outstanding Paper Prize for Research and Resource Articles published in 2023.

**Figure:**
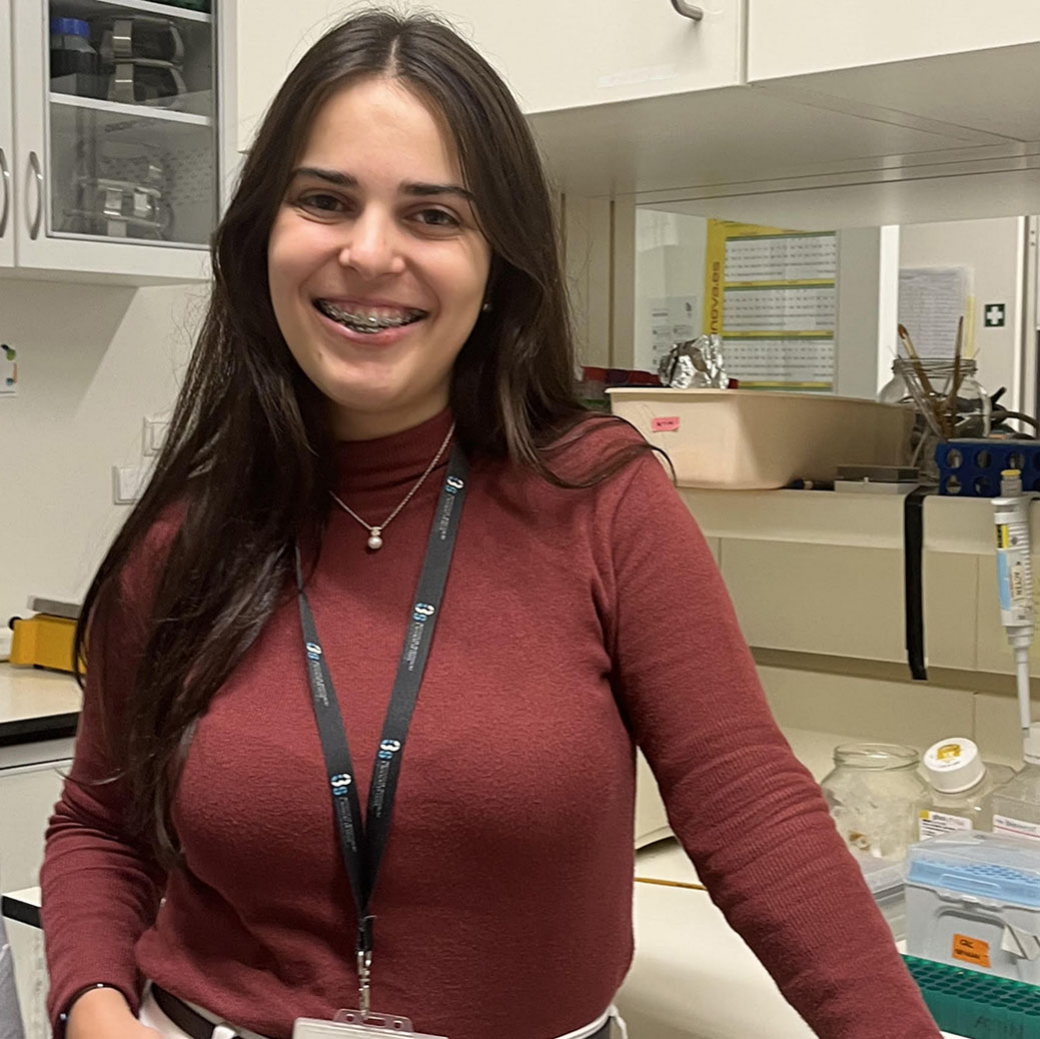
Lídia Faria

## Outstanding Paper Prize winner for Research Articles: Lídia Faria

Lídia is a young Portuguese researcher with expertise on breast cancer. Her scientific journey started in 2016, when she moved to Vila Real, Portugal and enrolled in a bachelor's degree in Genetics & Biotechnology. For three years, she gained a good foundation in biology and biotechnology. It was during this time that she became interested in exploring the underlying causes of cancer. She then worked as a trainee in the laboratory of Florence Janody, at i3S-Instituto de Investigação e Inovação em Saúde in Porto, Portugal, working to understand the interaction of actin cytoskeletal regulatory proteins with P-cadherin in breast cancer cellular models.

In 2019, she was accepted into the master's degree program in Oncology at the Portuguese Institute of Oncology of Porto (IPO Porto) and moved to Porto. During this time, she worked as a trainee in the Cytogenetics Laboratory at IPO Porto. In 2020, she joined the Cytoskeletal Regulation & Cancer lab at i3S, headed by Florence Janody, for her master's thesis, for which she obtained the maximum score of 20 in December 2021 from a jury composed of national and international researchers in the field. Her master's thesis work gave rise to a first author Research Article published in DMM (Faria et al., 2023), now awarded with the Outstanding Paper Prize. In this work, Lídia and colleagues generated a *Drosophila melanogaster* avatar expressing the cell-cell adhesion molecule P-cadherin, with the ultimate goal to uncover the role of P-cadherin-mediated cell signaling in benign breast lesions. The team validated their findings using a Src-inducible human mammary epithelial cell line, which recapitulates the molecular events taking place during malignant transformation. Their observations suggest that pre-malignant breast cells that will later progress to invasive cancer cells upregulate P-cadherin, which induces a boost of MRTF-SRF signaling through F-actin regulation.

In 2023, Lídia started her PhD under the supervision of Florence Janody, in collaboration with the laboratory of Anna Taubenberger at the CMCB-Center for Molecular and Cellular Bioengineering in Dresden, Germany, and the laboratory of Joana Paredes at i3S-Instituto de Investigação e Inovação em Saúde in Porto, Portugal, investigating the role of tension at adherens junctions in instructing the progression of pre-malignant breast cells to malignant cancer cells.

**Figure:**
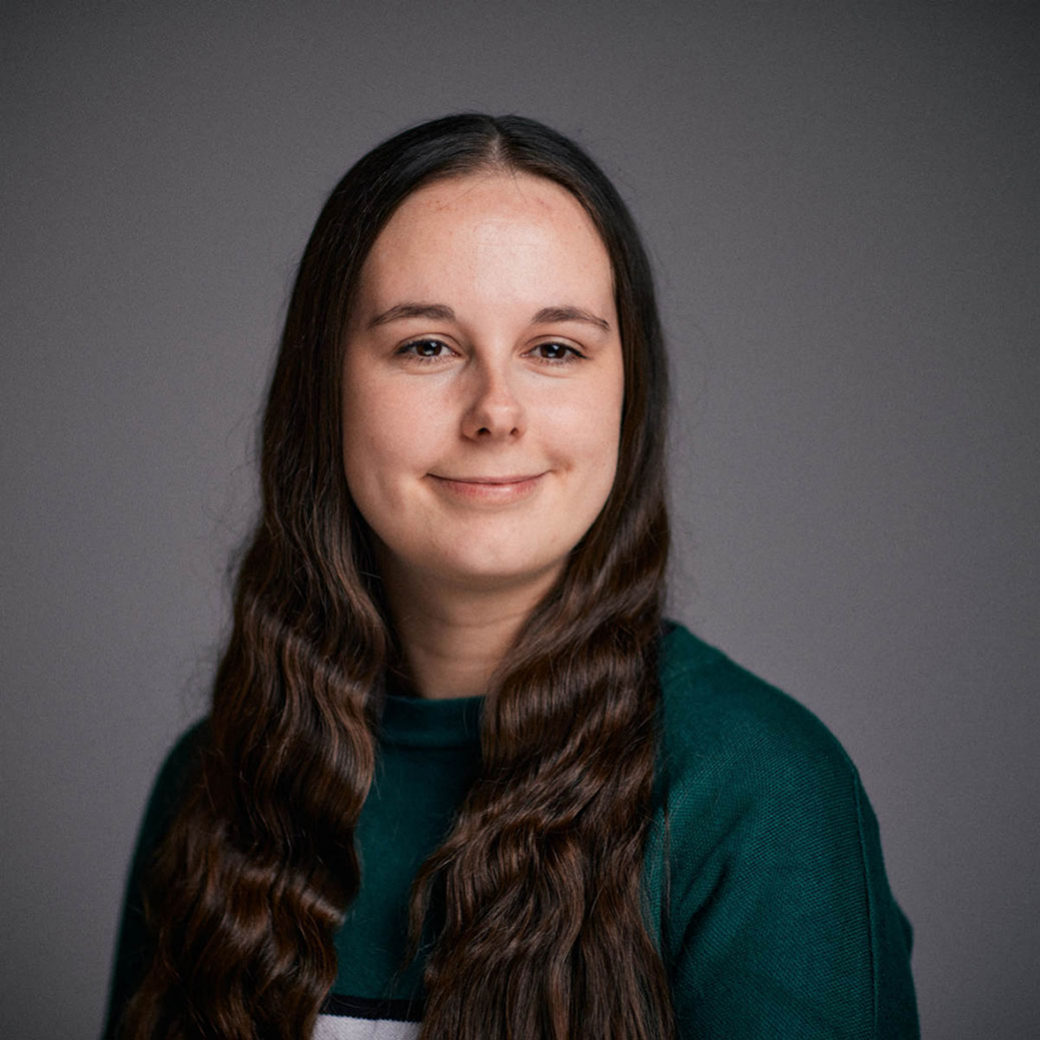
Ffion R. Hammond

## Outstanding Paper Prize winners for Resource Articles: Ffion R. Hammond and Amy Lewis

### Ffion Hammond

Ffion began her scientific career studying a BSc in biochemistry at Keele University, UK. During this time, Ffion developed an interest in immunology, which led her to undertake a placement year with the Cell Biology and Immunology (CBI) group at Wageningen University & Research (The Netherlands). This placement year involved a taught course module of human and veterinary immunology, and a research project that entailed characterising a zebrafish model of trypanosome infection, supervised by Dr Eva Dóró and Prof. Maria Forlenza. This project showcased the benefits of using the zebrafish model, because of their transparency and wide variety of genetic reporter lines, and sparked in Ffion an interest in both infection biology and the zebrafish model*.* This project combined high spatio-temporal resolution microscopy with the transparency of live zebrafish larvae to characterise parasite behaviours in the bloodstream, offering new insights into trypanosome infection dynamics.

Following her BSc, Ffion completed a short internship in glycobiology and mass spectrometry at Keele University with Dr Sarah Hart. This project aimed to use tandem mass spectrometric analyses to assess heparin sulphate disaccharide composition, and expanded Ffion's scientific understanding, areas of interest and technical skills into a new field of research.

However, Ffion's interest in infection biology and immunity continued into her PhD, conducted within the Department of Infection, Immunity and Cardiovascular disease (IICD) at the University of Sheffield, UK, supervised by Dr Philip Elks and Prof. Endre Kiss-Toth. The PhD also utilised the zebrafish model to understand host gene expression and the signalling pathways that influence infection dynamics, and how these might be manipulated to improve infection outcomes. Here, Ffion gained experience with multiple zebrafish infection models, including bacterial infections such as tuberculosis (TB; *Mycobacterium marinum* model) and *Staphylococcus aureus*, as well as fungal infections caused by *Cryptococcus neoformans* and *Candida albicans*. Each of these pathogens is associated with antimicrobial resistance (AMR), with both *C. neoformans* and *C. albicans* being added to the WHO fungal priority pathogens list. These pathogens are either resistant to antibiotics or – in the case of fungal pathogens – inherently more difficult to treat because of limited antifungal agents, and their eukaryotic nature makes it harder to target them without adverse side effects. This established Ffion's interest in alternative treatment routes to antimicrobials, and developed into aiming to understand host-derived or host-targeted approaches to fighting infection that can boost host immunity and circumvent the rise of AMR.

Following the completion of her PhD, Ffion began a postdoc position in immunity and AMR in September 2022, supervised by Prof. Dame Fiona Powrie at the Kennedy Institute of Rheumatology, University of Oxford, UK. As part of the Centre of Antimicrobial Research and Engineering (CARE), Ffion's current role investigates the interactions between AMR pathogens, the host microbiome and immune response, continuing her interest in host-directed therapeutics and addressing the global threat of AMR.

**Figure:**
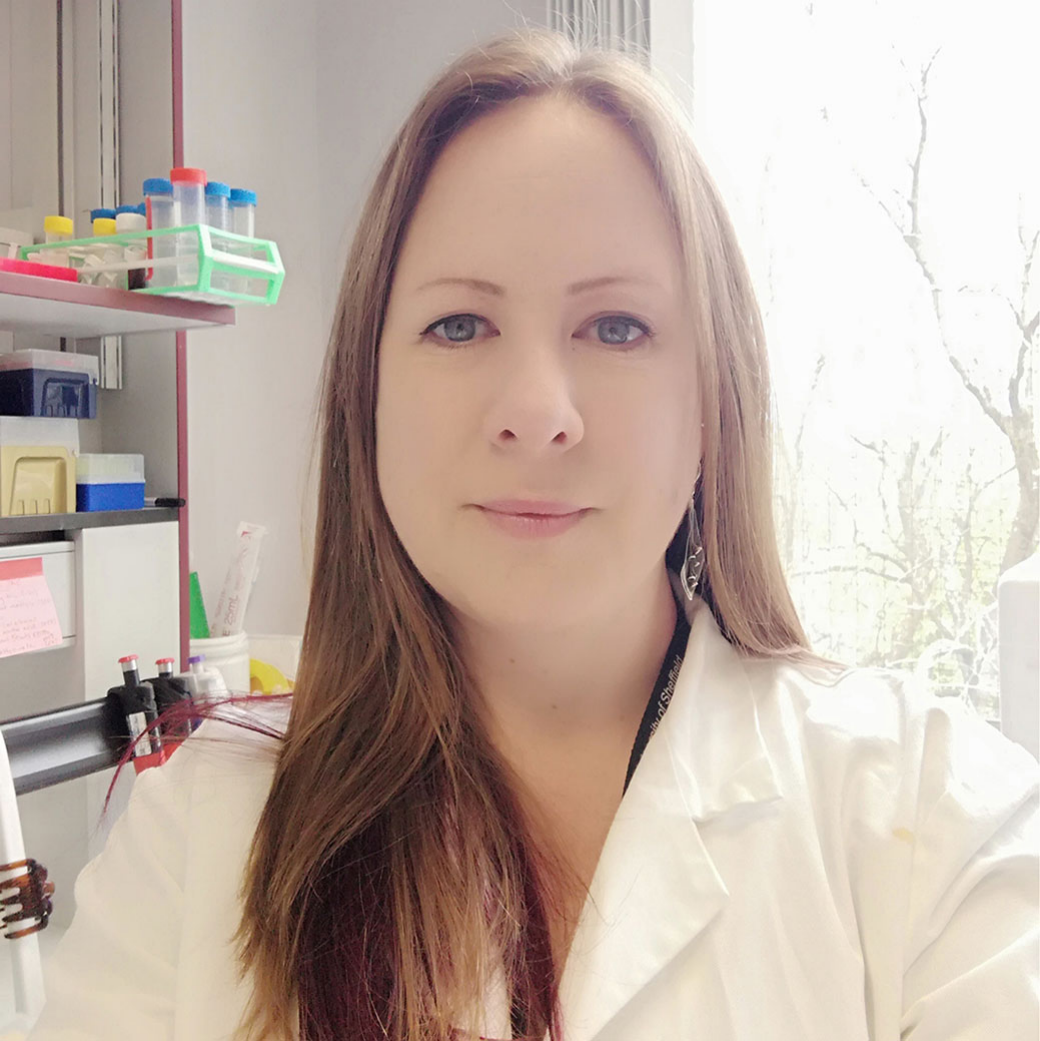
Amy Lewis

### Amy Lewis

Amy graduated with a BSc (Hons) in Immunology, during which time she undertook a year-long placement in the Department of Haematology at the Royal London Hospital, where she looked at intravenous immunoglobulin modulation of monocytes. Following an eight-year hiatus to work in local government in London, Amy gained an MSc in Biomedical Immunology. In 2006, she joined Dr Sandra Diebold’s lab, King's College London, UK, as a Research Assistant, studying the *in vivo* activation of dendritic cells with synthetic viral nucleic acids as adjuvants for tumour vaccines.

Following relocation to Sheffield, UK, Amy gained a post in Dr Peter Peachell’s lab, investigating IgE expression by pulmonary mast cells using resected lung tissue from cancer patients. She later moved to the Prof. Andy Heath lab, where she focused on the efficacy of CD40 monoclonal antibodies and TLR agonists as adjuvants in the prophylactic and therapeutic treatment of murine tumours.

Following this, she began working in the lab of Prof. Sarah Walmsley, looking at the regulation of inflammation via the neutrophil HIF pathway. Wishing to remain in Sheffield when the lab moved to Edinburgh, Amy undertook a year of maternity cover in Dr Simon Johnston's lab. During this time, she worked closely with Life Technologies to user-test their recently developed Attune NxT acoustic flow cytometer, with a view to using it to assess new therapeutics in a high-throughput *in vivo* zebrafish model of *C. neoformans*.

Amy enjoyed the challenges of working with zebrafish and, in 2015, she joined the newly established lab of Dr Phil Elks (School of Medicine and Population Health). Over the past nine years, Amy has focused on manipulation of neutrophil HIF as a therapeutic strategy for mycobacterial infection. Since joining the Elks lab, Amy has managed a move to a new purpose-built lab suite that includes a containment level 2 lab, and has been responsible for helping the lab expand to include numerous PhD, master’s and intercalating medical students. She has developed mutant and transgenic zebrafish lines to help better understand the spectrum of neutrophil activation states; this Outstanding Paper Prize was given to one such paper, which describes the arginase2:gfp zebrafish line.

Amy has also worked for several years to perfect a protocol for extracting RNA from sorted zebrafish neutrophils of sufficient quality to yield RNAseq data. In addition, she undertook some translational work using primary human neutrophils and skin biopsies taken from tuberculosis patients challenged with tuberculin protein.

During COVID, Amy volunteered her time to work alongside medical school colleagues processing samples for the ISARIC/WHO Characterisation of Severe Emerging Infections study, The Sheffield Teaching Hospitals HERO study and the Oxford/AstraZeneca vaccine trials.

**DMM Prize 2023 shortlist**

**Research Articles**

**A hepatoprotective role of peritumoral non-parenchymal cells in early liver tumorigenesis.**

Dis Cheng Tian, Liyuan Li, Li Fan, Anthony Brown, Eric J. Norris, Michelle Morrison, Evan S. Glazer and Liqin Zhu. *Dis. Model Mech.* (2023) 16, dmm049750. doi.org/10.1242/dmm.049750.

**Modeling the effects of genetic- and diet-induced obesity on melanoma progression in zebrafish.**

Emily Montal, Dianne Lumaquin, Yilun Ma, Shruthy Suresh and Richard M. White. *Dis. Model Mech.* (2023) 16, dmm049671. doi.org/10.1242/dmm.049671.

**Overexpression screen of chromosome 21 genes reveals modulators of Sonic hedgehog signaling relevant to Down syndrome.**

Anna J. Moyer, Fabian-Xosé Fernandez, Yicong Li, Donna K. Klinedinst, Liliana D. Florea, Yasuhiro Kazuki, Mitsuo Oshimura and Roger H. Reeves. *Dis. Model Mech.* (2023) 16, dmm049712. doi.org/10.1242/dmm.049712.

**Effectiveness of irinotecan plus trabectedin on a desmoplastic small round cell tumor patient-derived xenograft.**

Valentina Zuco, Sandro Pasquali, Monica Tortoreto, Stefano Percio, Valentina Doldi, Marta Barisella, Paola Collini, Gian Paolo Dagrada, Silvia Brich, Patrizia Gasparini, Marco Fiore, Michela Casanova, Anna Maria Frezza, Alessandro Gronchi, Silvia Stacchiotti, Andrea Ferrari, Nadia Zaffaroni. *Dis. Model Mech.* (2023) 16, dmm049649. doi.org/10.1242/dmm.049649.

**Maternal heterozygosity of Slc6a19 causes metabolic perturbation and congenital NAD deficiency disorder in mice.**

Hartmut Cuny, Kayleigh Bozon, Rosemary B. Kirk, Delicia Z. Sheng, Stefan Bröer, Sally L. Dunwoodie. *Dis. Model Mech.* (2023) 16, dmm049647. doi.org/10.1242/dmm.049647.

**Multiplatform modeling of atrial fibrillation identifies phospholamban as a central regulator of cardiac rhythm.**

Anaïs Kervadec, James Kezos, Haibo Ni, Michael Yu, James Marchant, Sean Spiering, Suraj Kannan, Chulan Kwon, Peter Andersen, Rolf Bodmer, Eleonora Grandi, Karen Ocorr, Alexandre R. Colas. *Dis. Model Mech.* (2023) 16, dmm049962. doi.org/10.1242/dmm.049962.

**A novel porcine model of CLN3 Batten disease recapitulates clinical phenotypes.**

Vicki J. Swier, Katherine A. White, Tyler B. Johnson, Xiaojun Wang, Jimin Han, David A. Pearce, Ruchira Singh, Arlene V. Drack, Wanda Pfeifer, Christopher S. Rogers, Jon J. Brudvig, Jill M. Weimer. *Dis. Model Mech.* (2023) 16, dmm050038. doi.org/10.1242/dmm.050038.

**Activation of an actin signaling pathway in pre-malignant mammary epithelial cells by P-cadherin is essential for transformation.**

Lídia Faria, Sara Canato, Tito T. Jesus, Margarida Gonçalves, Patrícia S. Guerreiro, Carla S. Lopes, Isabel Meireles, Eurico Morais-de-Sá, Joana Paredes, Florence Janody. *Dis. Model Mech.* (2023) 16, dmm049652. doi.org/10.1242/dmm.049652.

**HuR modulation counteracts lipopolysaccharide response in murine macrophages.**

Isabelle Bonomo, Giulia Assoni, Valeria La Pietra, Giulia Canarutto, Elisa Facen, Greta Donati, Chiara Zucal, Silvia Genovese, Mariachiara Micaelli, Anna Pérez-Ràfols, Sergio Robbiati, Dimitris L. Kontoyannis, Marilenia De Matteo, Marco Fragai, Pierfausto Seneci, Luciana Marinelli, Daniela Arosio, Silvano Piazza, Alessandro Provenzani. *Dis. Model Mech.* (2023) 16, dmm050120. doi.org/10.1242/dmm.050120.

**A *Drosophila* chemical screen reveals synergistic effect of MEK and DGKα inhibition in Ras-driven cancer.**

John E. La Marca, Robert W. Ely, Sarah T. Diepstraten, Peter Burke, Gemma L. Kelly, Patrick O. Humbert, Helena E. Richardson. *Dis. Model Mech.* (2023) 16, dmm049769. doi.org/10.1242/dmm.049769.

**Resource articles**

**Tellu: an object detector algorithm for automatic classification of intestinal organoids.**

Eva Domenech-Moreno, Anders Brandt, Toni T Lemmetyinen, Linnea Wartiovaara, Tomi P Makela, and Saara Ollila. *Dis. Model Mech.* (2023) 16, dmm049756.doi.org/10.1242/dmm.049756.

**Efficient genetic editing of human intestinal 1 organoids using ribonucleoprotein-based CRISPR.**

Nefeli Skoufou-Papoutsaki, Sam Adler, Paula D'Santos, Liz Mannion, Shenay Mehmed, Richard Kemp, Amy Smith, Francesca Perrone, Komal Nayak, Alasdair Russell, Matthias Zilbauer, and Douglas J Winton. *Dis. Model Mech.* (2023) 16, dmm050279. doi.org/10.1242/dmm.050279.

**Winner: Research Article**

**Activation of an actin signaling pathway in pre-malignant mammary epithelial cells by P-cadherin is essential for transformation.**

Lídia Faria, Sara Canato, Tito T. Jesus, Margarida Gonçalves, Patrícia S. Guerreiro, Carla S. Lopes, Isabel Meireles, Eurico Morais-de-Sá, Joana Paredes, Florence Janody. *Dis. Model Mech.* (2023) 16, dmm049652. doi.org/10.1242/dmm.049652.

**Winner: Resource Article**

**An arginase 2 promoter transgenic line illuminates immune cell polarisation in zebrafish.**

Hammond, F. R., Lewis, A., Speirs, Z. C., Anderson, H. E., Sipka, T., Williams, L. G., Nguyen-Chi, M., Meijer, A. H., Wiegertjes, G. F. and Elks, P. M. (2023**).** *Dis. Model Mech.* (2023) 16, dmm049966. doi.org/10.1242/dmm.049966.
